# Perfusion Patterns of Pleura-Based Pulmonary Sarcoma Metastases on Contrast-Enhanced Ultrasound (CEUS): A Single-Center Retrospective Pilot Study

**DOI:** 10.3390/diagnostics16111706

**Published:** 2026-06-02

**Authors:** Felix Ragnar Merlin Koenig, Christian Görg, Helmut Prosch, Veronika Vetchy, Anna Hohensteiner, Nikolai A. Gantner, Daria Kifjak, Florian Lindenlaub, Philipp Theodor Funovics, Iris-Melanie Noebauer-Huhmann, Ehsan Safai Zadeh

**Affiliations:** 1 Department of Biomedical Imaging and Image Guided Therapy, Medical University of Vienna, 1090 Vienna, Austriaehsan.safaizadeh@meduniwien.ac.at (E.S.Z.); 2 High Field Magnetic Resonance Center, Department of Biomedical Imaging and Image-Guided Therapy, Medical University of Vienna, 1090 Vienna, Austria; 3 Interdisciplinary Center of Ultrasound Diagnostics, Haematology, Oncology and Immunology, University Hospital Giessen and Marburg, Philipps University Marburg, Baldingerstraße, 35037 Marburg, Germany; 4 Department of Orthopedics and Trauma-Surgery, Medical University of Vienna, 1090 Vienna, Austria

**Keywords:** soft-tissue sarcoma, contrast-enhanced ultrasound, lung ultrasound, vascularization

## Abstract

**Background/Objectives:** Pleura-based pulmonary nodules in soft-tissue sarcoma (STS) patients remain diagnostically challenging, and entity-specific contrast-enhanced ultrasound (CEUS) data are scarce. We aimed to characterize CEUS perfusion patterns of pleura-based STS pulmonary metastases in a pilot cohort. **Methods:** We investigated a single-center retrospective cohort at a tertiary STS referral center (Dec 2024–Dec 2025). Of 51 consecutive STS patients with suspected pulmonary metastases screened, 32 lacked pleural contact and 6 were excluded for logistical reasons; the remaining 13 underwent standardized CEUS of a pleura-contacting lesion (≥5 mm) visible on B-mode lung ultrasound (B-LUS), with 1 excluded on biopsy (anaplastic lymphoma). The reference standard combined histology, therapy-related size reduction of the index lesion, and/or documented distant metastatic STS. Two readers rated all examinations independently, with adjudication by a third senior reader. Wilson 95% confidence intervals (CIs) and Cohen’s κ were computed. **Results:** In the 12 analyzed patients (mean age 58.8 ± 17.8 years; 7 male), the index lesion was histologically confirmed in 4 (33.3%). On CEUS, bronchial-arterial (BA) enhancement predominated (10/12; 83.3%, 95% CI 55.2–95.3%) and pulmonary-arterial timing occurred in 2/12 (16.7%). Marked enhancement was present in 9/12 (75.0%), homogeneous in 8/12 (66.7%), and rapid washout (<120 s) in all lesions (12/12; 100%, 95% CI 75.8–100%). Inter-reader agreement was substantial to almost perfect for the diagnostically relevant CEUS perfusion variables (enhancement κ = 0.75; EE κ = 0.80; HE κ = 0.82) and moderate for the descriptive shape variable (Form κ = 0.47). **Conclusions:** In this selected pilot cohort, pleura-based STS lung metastases most commonly showed BA-dominant enhancement with universal rapid washout. The findings are hypothesis-generating and require validation in larger, prospective multicenter cohorts.

## 1. Introduction

In patients with a known malignancy presenting with newly detected pulmonary nodules, metastatic disease must be considered a key differential diagnosis [1]. Chest computed tomography (CT) represents the first-line diagnostic modality; however, diagnostic uncertainty frequently persists, and in cases with suspicion of malignancy, definitive diagnosis is ultimately established by histologic confirmation [2]. B-mode lung ultrasound (B-LUS) has been used for several years as an imaging modality for the detection of pleural effusion [3,4], guidance of thoracentesis [5], and ultrasound-guided thoracic interventions [6], as well as for the visualization of pleura-adjacent pulmonary lesions [7].

However, in the assessment of malignancy, conventional B-LUS has limited diagnostic value. In selected clinical scenarios and with a targeted diagnostic question, evaluation of lesion vascularization may provide additional insight. In this setting, contrast-enhanced ultrasound (CEUS) can provide additional, real-time information on lesion vascularity and perfusion patterns, thereby increasing diagnostic confidence [8]. Given the lung’s dual arterial supply (pulmonary vs. bronchial) [9], benign entities such as pneumonia typically show a pulmonary-arterial (PA) enhancement pattern, whereas invasive processes—including malignant tumors—more often demonstrate bronchial-arterial (BA) enhancement on CEUS [8]. This principle, however, should not be applied to all malignant lesions. It has been demonstrated that certain benign chronic processes, such as organizing pneumonia and granulomatous diseases, may exhibit a BA enhancement pattern [10,11]. Conversely, specific malignant pulmonary entities, including pulmonary lymphomas, can be characterized by a predominantly PA perfusion pattern on CEUS [12]. Therefore, it is essential to investigate each entity individually and to characterize the perfusion patterns of specific entities separately. With regard to soft tissue sarcomas (STSs), data on CEUS perfusion characteristics remain scarce. The aim of this study was to describe CEUS perfusion patterns of subpleural pulmonary STS metastases in a consecutive cohort from a tertiary sarcoma referral center.

## 2. Methods

This single-center retrospective cohort study was conducted at a tertiary sarcoma referral center. Between December 2024 and December 2025, consecutive patients with histologically confirmed STS and suspected pulmonary metastases discussed at the Sarcoma Tumor Board were identified retrospectively from clinical records. During routine care, CEUS had been performed solely for clinical indications upon referral from the treating sarcoma outpatient clinic of the Department of Orthopedic Surgery, namely for short-term follow-up under ongoing therapy and to correlate peripheral pleura-based lesions with cross-sectional imaging findings (CT or magnetic resonance imaging [MRI]). All CEUS examinations followed the department’s standard operating procedure and were carried out by a single investigator with 10 years of dedicated CEUS experience under the supervision of an “Österreichische Gesellschaft für Ultraschall in der Medizin” (ÖGUM) Level III-certified expert. Written informed consent was obtained for the clinical CEUS examination. The retrospective analysis of pseudonymized data was approved by the local ethics committee (EK: 2143/2025–16 December 2025) and conducted in accordance with the Declaration of Helsinki.

Eligibility criteria for this retrospective analysis followed established CEUS lung protocols and required all of the following:

- Adults (≥18 years) who underwent CEUS of a subpleural pulmonary lesion;
- Pleura-adjacent location of the lesion such that B-LUS visualization and CEUS examination were feasible, with a lesion long axis of at least 5 mm (0.5 cm) on B-LUS;
- Standardized documentation of B-LUS and CEUS cine loops/still frames;
- An available reference standard, defined as (a) histologically confirmed pulmonary sarcoma metastasis, (b) documented size reduction of the index lesion under sarcoma-directed systemic therapy, or (c) a new pulmonary lesion in a patient with histologically proven distant metastasis of sarcoma.

Exclusion criteria were: lack of pleural contact, inability to perform CEUS for logistical/clinical reasons (e.g., travel constraints, Intensive Care Unit (ICU) care, patient not reachable via the treating orthopedist), or a non-sarcoma final diagnosis on biopsy.

**Figure 1:**
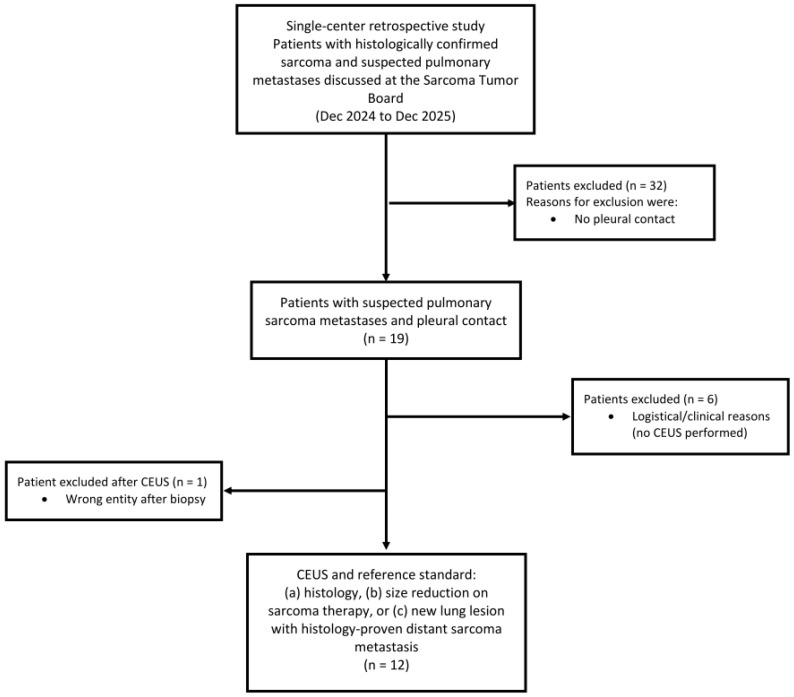
Study flow for the single-center, retrospective cohort (Dec 2024–Dec 2025).

During the screening window (Dec 2024–Dec 2025), 51 tumor-board cases with histologically confirmed STS and suspected pulmonary metastasis were screened. Of these, 32 lesions had no pleural contact and were therefore not amenable to B-LUS/CEUS, leaving 19 patients with a suspected pleura-based pulmonary lesion. Of these 19, 6 were excluded for logistical or clinical reasons (e.g., travel constraints, Intensive Care Unit care, patient not reachable via the treating orthopedist). The remaining 13 patients underwent CEUS according to the standardized protocol. After subsequent biopsy, one case was excluded because the pulmonary lesion proved to be a pulmonary manifestation of an anaplastic lymphoma rather than a sarcoma metastasis, yielding a final CEUS analysis cohort of *n* = 12 (Figure 1).

### 2.1. Ultrasound

All B-LUS and CEUS examinations were performed with a GE LOGIQ E10 (GE Healthcare, Chicago, IL, USA) using a curved-array transducer (low-frequency, 1–5 MHz) on a high-end ultrasound platform in contrast-specific mode for CEUS, in accordance with European Federation of Societies for Ultrasound in Medicine and Biology (EFSUMB) recommendations [13,14]. CEUS used an intravenous bolus of 2.4 mL sulfur-hexafluoride microbubbles (SonoVue^®^, Bracco Imaging, Milan, Italy) followed by a 10 mL 0.9% NaCl flush. Imaging was acquired in the sitting position, with intercostal access parallel to the ribs. After contrast injection, continuous cine acquisition covered the early arterial inflow (0–60 s), followed by re-evaluation at 1 and 2–3 min with clips and still images archived. All examinations were standardized and performed by the same investigator (10 years thoracic CEUS experience).

### 2.2. B-Mode Lung Ultrasound Parameters

B-LUS features were extracted retrospectively from the recorded loops/stills and coded according to the study worksheet:

- Form—overall shape of the pleura-based lesion (round/oval).
- Boundary—lesion margin characteristics at the pleural interface (Well-Circumscribed/Ill-defined).
- Main_echo—echogenicity of the lesion relative to adjacent thoracic wall/parenchymal reference (hypoechoic/isoechogenic/hyperechoic).
- Size—in-plane lesion dimensions measured as long axis and short axis (cm). The maximum diameter is reported as the long axis; the short axis is documented for shape/aspect-ratio.

Only pleura-based lesions (long axis ≥ 0.5 cm) that were visualizable by B-LUS were considered eligible for CEUS analysis.

### 2.3. Contrast-Enhanced Ultrasound Parameters

CEUS perfusion was categorized with the following variables:

- Time to Enhancement (TE)—enhancement timing after injection, dichotomized into
  ○ Early PA enhancement: lesion enhancement before visible enhancement in the chest wall (intercostal artery) used as the in vivo systemic reference, indicating a predominant pulmonary-arterial supply;
  ○ Delayed BA enhancement: lesion enhancement simultaneously with, or after, contrast arrival in the chest-wall (intercostal-artery) reference, indicating a predominant systemic bronchial-arterial supply, in line with the chest-wall reference approach previously described in lung CEUS.
- Extent of Enhancement (EE)—arterial-phase intensity relative to an in vivo reference (e.g., spleen/thoracic wall): reduced (hypoechoic) vs. marked (iso-echogenic).
- Homogeneity of Enhancement (HE)—spatial enhancement pattern during the arterial phase: homogeneous vs. inhomogeneous; lesions with co-existing non-perfused areas (NPAs) were coded as inhomogeneous.
- Decrease in Enhancement (DE)—washout behavior in the parenchymal phase: rapid washout (<120 s) vs. late washout (≥120 s).

### 2.4. Reading Procedure and Data Handling

Two experienced investigators independently reviewed all B-LUS and CEUS datasets using a standardized case report form aligned with the predefined worksheet variables. Both readers were aware of the underlying STS diagnosis and of the clinical indication for CEUS, but were blinded to each other’s ratings; cross-sectional imaging reports were available, but the readers did not have access to histological results when scoring CEUS variables. In patients with multiple pulmonary nodules, the index lesion was prospectively defined at the time of the clinical CEUS examination as the largest pleura-contacting lesion that was reliably visualized on B-LUS in a single intercostal window; only this single index lesion per patient was scored. Discordances between the two primary readers occurred in 6/84 ratings (7.1%) and were limited to four variables: lesion shape (Form, 3 cases), enhancement timing (PA vs. BA, 1 case), extent of enhancement (1 case), and homogeneity of enhancement (1 case); none of the cases of zero-variance variables (echogenicity, boundary, decrease in enhancement) showed discordance. All discordances were adjudicated by a third senior expert and finalized by consensus. Clinical data (age, sex, sarcoma subtype, and therapy) and cross-sectional imaging findings (MRI; CT and Positron Emission Tomography–Computed Tomography (PET-CT) characteristics; lesion size, when available) were extracted from the electronic medical records. All data were pseudonymized prior to statistical analysis. For each patient, the time interval between the most recent prior chest CT and the CEUS examination was recorded.

### 2.5. Statistical Analysis

Given the small, hypothesis-generating nature of this pilot cohort and the prespecified categorical CEUS variables, the analysis was descriptive. Continuous variables are reported as mean ± standard deviation (range), and categorical variables as absolute and relative frequencies. For each key categorical proportion, two-sided 95% confidence intervals (CIs) were calculated using the Wilson score method, which is preferred over the normal approximation for small samples and for proportions near 0% or 100%. Inter-reader agreement between the two primary readers was quantified per categorical variable as observed agreement (P_o_) and Cohen’s κ, with Landis–Koch interpretation (<0.21 slight, 0.21–0.40 fair, 0.41–0.60 moderate, 0.61–0.80 substantial, 0.81–1.00 almost perfect); for variables with zero observed variance across the cohort, κ is undefined and only P_o_ is reported. No formal hypothesis testing or comparison of CEUS patterns between histological subtypes was performed, because the cohort size of *n* = 12 does not provide adequate statistical power for such inference; reported percentages should therefore be interpreted as exploratory point estimates with the corresponding interval ranges, rather than as precise diagnostic-performance estimates. Data were processed in Microsoft Excel (Microsoft Corporation, Redmond, WA, USA); CI and Cohen’s κ were computed using a Wilson score implementation and the cohen_kappa_score function in Python 3.14.3 (SciPy, scikit-learn, Python Software Foundation, Wilmington, DE, USA).

## 3. Results

### 3.1. Patient Cohort

**Figure 2:**
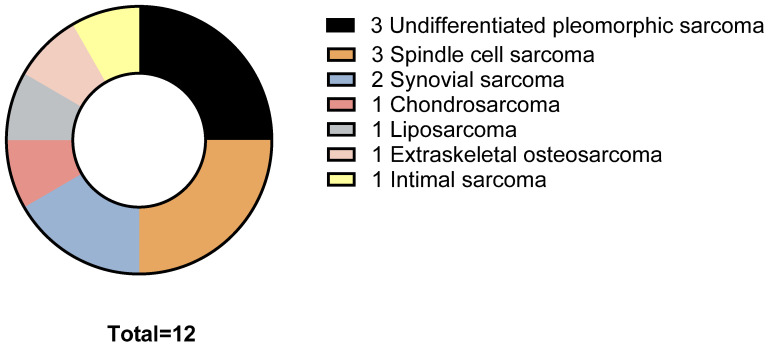
Distribution of primary STS histologic subtypes in the study cohort (*n* = 12).

Among the 12 patients included in the analysis, 7 were male and 5 were female. The mean age at examination was 58.8 ± 17.8 years (range, 29–85 years). All patients had histologically confirmed primary STS. The predominant histologic subtypes were undifferentiated pleomorphic sarcoma (*n* = 3) and spindle cell sarcoma (*n* = 3), followed by synovial sarcoma (*n* = 2). Single cases of intimal sarcoma, liposarcoma, chondrosarcoma, and extraskeletal osteosarcoma were also represented (Figure 2). Malignancy of the pulmonary lesion was confirmed by biopsy and/or surgical resection in four patients (33.3%). In seven patients (58.3%), the metastatic nature of the lesion was established on the basis of histologically confirmed distant metastasis and a marked reduction in the size of the index lesion under sarcoma-directed systemic therapy. The remaining patient (8.3%) presented with a newly detected pulmonary lesion in the context of previously documented metastatic STS, consistent with metastatic disease. In all patients, chest CT served as the comparator cross-sectional imaging study; the median interval between chest CT and CEUS was 18 days (IQR 5–36 days; range 0–76 days).

### 3.2. B-Mode Ultrasound Data

All 12 lesions appeared hypoechoic on baseline B-LUS. All lesions had well-circumscribed margins on ultrasound; eight lesions (66.7%) were oval in shape and the remaining four lesions (33.3%) were round. The average lesion size on B-mode was approximately 2.6 × 1.7 cm, with a range from 0.7 × 0.6 cm (smallest lesion) to 7.6 × 5.5 cm (largest lesion).

### 3.3. Contrast-Enhanced Ultrasound Data

**Figure 3:**
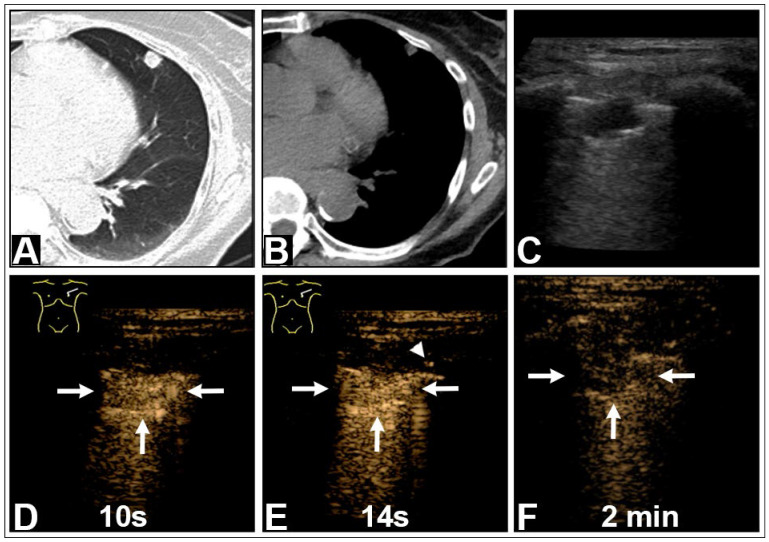
Representative biopsy-confirmed case of a 75-year-old female patient with a diagnosis of spindle cell sarcoma and pulmonary nodules on CT (**A**,**B**). On B-mode ultrasound (**C**), a hypoechoic pleura-adjacent pulmonary nodule was identified. On CEUS (**D**), the lesion demonstrated early enhancement after 10 s (arrows), preceding enhancement of the thoracic wall vessels, which became visible only after 14 s ((**E**), arrowhead). The lesion exhibited a marked and homogeneous enhancement pattern, consistent with a predominantly pulmonary-arterial supply. At approximately 2 min (**F**), an early decline in enhancement was observed, indicating rapid washout.

**Figure 4:**
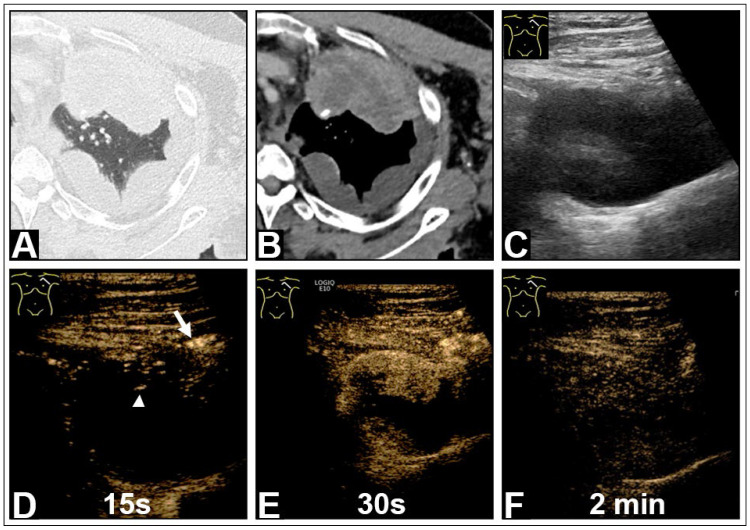
63-year-old male patient with a known diagnosis of undifferentiated pleomorphic sarcoma and multiple pulmonary lesions on CT (**A**,**B**). On B-mode ultrasound (**C**), a hypoechoic pleura-adjacent pulmonary nodule was identified. On CEUS (**D**), the lesion (arrowhead) demonstrated a bronchial-arterial enhancement pattern, with enhancement occurring simultaneously with the arrival of contrast in the intercostal artery (arrows). The lesion exhibited marked and inhomogeneous enhancement (**E**), followed by an early decrease in enhancement at approximately 2 min (**F**), consistent with rapid washout.

On CEUS, two lesions (16.7%; 95% CI 4.7–44.8%) exhibited an early PA enhancement pattern (Figure 3), whereas the majority, 10 lesions (83.3%; 95% CI 55.2–95.3%), demonstrated a delayed BA enhancement pattern (Figure 4).

Furthermore, nine lesions (75.0%; 95% CI 46.8–91.1%) showed marked contrast enhancement, while three lesions (25.0%; 95% CI 8.9–53.2%) exhibited reduced enhancement intensity. The enhancement was homogeneous in eight lesions (66.7%; 95% CI 39.1–86.2%) and inhomogeneous in the remaining four lesions (33.3%; 95% CI 13.8–60.9%). Additionally, all 12 lesions (100%; 95% CI 75.8–100%) displayed a rapid contrast washout (<120 s), with none of the lesions showing a late washout phase. The proportion of lesions with very early washout (<60 s), as has been described in some studies of high-grade malignancy, was not formally pre-specified in the worksheet and is therefore not separately reported here; this represents an avenue for prospective evaluation in future studies. Inter-reader agreement between the two primary readers was substantial to almost perfect for the diagnostically relevant CEUS perfusion variables (enhancement P_o_ = 0.92, κ = 0.75, substantial; EE P_o_ = 0.92, κ = 0.80, substantial; HE P_o_ = 0.92, κ = 0.82, almost perfect) and moderate for the descriptive shape variable form (P_o_ = 0.75, κ = 0.47, moderate). Echogenicity, boundary, and decrease in enhancement showed 100% observed agreement; κ is undefined for these variables because all 12 lesions received identical ratings (no observed variance). Across all 84 reader-by-variable ratings, 78 (92.9%) were concordant; the remaining six discordances were resolved by adjudication by the third senior expert.

## 4. Discussion

STSs are rare malignancies, accounting for approximately 1% of all adult cancers [15,16]. The lung is the most common—and clinically most relevant—site of distant spread: depending on histologic subtype and primary tumor location (e.g., extremity vs. trunk), approximately 10–40% of patients develop pulmonary metastases during the course of disease [17,18]. These observations are consistent with the number of patients identified during the study period. Despite the limited sample size, our findings contribute to addressing a recognized evidence gap in CEUS practice, as lung CEUS remains insufficiently studied and is supported by relatively sparse patient-level data [13]. By focusing on pleura-based sarcoma metastases, this study offers a rare glimpse into CEUS behavior in this subgroup, contributing novel data where guidelines and prior studies have identified a paucity of evidence [14].

In this study, pulmonary sarcoma metastases predominantly demonstrated a BA perfusion pattern with frequently marked and predominantly homogeneous enhancement and rapid washout. Specifically, 10 of 12 metastases (83.3%) exhibited a BA perfusion pattern, and all lesions demonstrated rapid washout (<120 s). This pattern is expected in neoplastic lesions characterized by tumor-associated neoangiogenesis [19,20]. The pulmonary arterial system has limited intrinsic angiogenic capacity [21,22]. In contrast, lesions associated with parenchymal destruction, such as non-hematologic malignant pulmonary lesions, including primary lung cancer and non-hematologic metastases, as well as certain chronic inflammatory conditions (e.g., granulomatous disease and organizing pneumonia), typically demonstrate BA vascularization [10,11].

The early PA enhancement pattern, observed in a minority of cases, may be explained by the presence of arteriovenous or bronchopulmonary shunts, allowing pulmonary arterial perfusion to reach the lesion before bronchial arterial inflow becomes dominant or clearly demarcated. Rapid washout may reflect extensive pathological shunting and can be interpreted as a surrogate marker of aberrant angiogenesis, thereby constituting a potential warning sign of underlying vascular remodeling [23,24].

Furthermore, 9 of 12 lesions (75.0%) exhibited marked enhancement and 8 of 12 (66.7%) demonstrated homogeneous enhancement on CEUS. The HE and EE likely reflect the extent of neoangiogenesis and the presence or absence of intralesional necrosis. However, these parameters were heterogeneous across the study population and, in isolation, cannot be reliably used as definitive criteria of malignancy [11].

A recent meta-analysis reported a pooled sensitivity of 95% and specificity of 93% for CEUS in differentiating malignant versus benign subpleural lung lesions [25]. Such performance is achieved by evaluating multiple contrast features in combination—for example, integrating enhancement timing, intensity, and homogeneity improves discrimination of malignancy [25]. Nevertheless, the binary categorization of “benign” versus “malignant” highlights the considerable overlap between entities (Table 1). Therefore, histologic confirmation through biopsy remains essential in clinically relevant scenarios, particularly to secure entity-specific diagnosis and to ensure accurate lesion characterization.

Prior studies have confirmed that performing biopsies with CEUS planning significantly increases tissue adequacy and accuracy [29]. From a treatment planning perspective, enhancement patterns may also be clinically valuable: highly vascular (marked enhancement) might respond differently to anti-angiogenic therapies or perfusion-targeted treatments than poorly perfused lesions [30,31]. By visualizing microvascularization in real time, CEUS could therefore complement oncologic decision-making [14]. For example, homogeneous, intensely enhancing metastases may indicate robust perfusion that supports systemic delivery [32], whereas heterogeneous enhancement with non-perfused areas may suggest necrosis and could inform targeting of viable tissue for loco-regional approaches (e.g., ablation) [33]. While speculative, these considerations illustrate the potential clinical utility of CEUS findings beyond diagnosis. Our conservative interpretation avoids overstating these possibilities, but it is forward-looking: as more evidence accrues, CEUS perfusion characteristics might guide personalized management of metastatic lung lesions.

In selected scenarios, CEUS may complement—not replace—standard CT: (i) bedside interrogation of pleura-based nodules that remain indeterminate on CT, as illustrated by the anaplastic-lymphoma case in this cohort; (ii) radiation-free interim follow-up of selected pleura-based index lesions between CT studies during sarcoma-directed therapy; and (iii) real-time guidance of transthoracic biopsy toward viable, perfused tissue. These use cases require prospective validation before any guideline-level recommendation.

### Strengths and Limitations

Several limitations of this pilot study should be made explicit, and they shape the interpretation of the present findings. First, the cohort comprised only 12 patients with one index lesion each. Although consistent with the rarity of pleura-based STS lung metastases at a tertiary referral center over a 12-month window, the small sample size yields wide 95% CI around all proportions and precludes formal subgroup analysis by histological subtype or therapy. Second, the cohort represents a highly selected subgroup of pulmonary STS lesions: only lesions with pleural contact that were reliably visible on B-LUS were eligible, while non-pleural-contacting nodules—which represent the majority of pulmonary metastases overall—were not assessable by CEUS by definition. The findings therefore apply specifically to pleura-based, B-LUS-visible STS metastases and should not be extrapolated to peripheral or non-pleural-contacting nodules in general. Third, the reference standard was heterogeneous: histological confirmation of the index lesion by biopsy and/or surgical resection was available in 4/12 (33.3%), while the remaining cases were classified on the basis of histologically proven distant sarcoma metastasis combined with documented size reduction of the index lesion under sarcoma-directed systemic therapy (7/12, 58.3%) or a newly detected pulmonary lesion in a patient with previously documented metastatic STS (1/12, 8.3%). Although this composite standard is a pragmatic compromise mirroring sarcoma-board practice (universal biopsy of subpleural lesions in proven metastatic STS being rarely indicated and not retrospectively available), it is less robust than universal histological confirmation. Fourth, although Cohen’s κ was substantial to almost perfect for the diagnostically relevant CEUS perfusion variables (enhancement κ = 0.75; EE κ = 0.80; HE κ = 0.82), agreement was only moderate for the lesion-shape variable form (κ = 0.47 P_o_ = 0.75). The lower κ for form should be interpreted in light of the well-described kappa paradox at small sample sizes with skewed marginal distributions, the inherent subjectivity of an oval-versus-round dichotomy, and the fact that lesion shape carries no direct diagnostic CEUS information; we therefore did not modify the interpretation of the perfusion findings on this account. Fifth, the study is single-center and involved a single highly experienced operator under expert supervision, which optimizes acquisition quality but limits external generalizability and does not allow for assessment of operator dependency. Sixth, no quantitative time–intensity curve analysis was performed; the present analysis is restricted to the established categorical EFSUMB-aligned descriptors (TE, EE, HE, DE), with quantitative dynamic CEUS reserved for future prospective work. Strengths include the consecutive enrollment from a clearly defined sarcoma-board pathway at a tertiary referral center, the standardized acquisition and worksheet-based reading procedure aligned with EFSUMB recommendations, the entity-specific focus on a tumor group for which dedicated CEUS lung data have been explicitly identified as lacking, the formal quantification of inter-reader agreement, and the favorable safety profile of CEUS contrast agents (reported serious adverse-event rates of approximately 0.0086%), which is particularly relevant for STS patients undergoing repeated follow-up imaging.

Finally, we emphasize a forward-looking but cautious outlook. This dataset helps to fill a small knowledge gap in CEUS, demonstrating that even rare entities such as STS lung metastases largely adhere to the vascular patterns observed in more prevalent pulmonary malignancies. In doing so, it offers a path toward a more pattern-based evaluation of lung lesions with CEUS that integrates enhancement timing, intensity, and texture to improve diagnostic specificity. At the same time, we remain conservative in our conclusions. We do not propose CEUS as a standalone replacement for established modalities, but rather as a powerful adjunct that can enhance diagnostic confidence in experienced hands. In line with EFSUMB recommendations, any CEUS assessment must be interpreted in the context of clinical presentation and the imaging picture [13]. Our study contributes to that picture by describing, in a small but consecutively enrolled and entity-specific cohort, what pleura-based, B-LUS-visible STS metastases tend to look like on CEUS. The potential clinical implications—more confident bedside identification of pleura-based lesions consistent with metastasis, more informed selection of lesions and trajectories for transthoracic biopsy, and contrast-agent-based monitoring of selected lesions during follow-up of metastatic STS—should be regarded as hypotheses generated by this pilot rather than as established performance claims. In conclusion, this retrospective pilot cohort—explicitly limited by its small sample size, by its single-center, single-operator setting, and by a heterogeneous reference standard—describes preliminary CEUS perfusion patterns of pleura-based STS lung metastases and motivates a larger, multicenter prospective study, with a universal histological reference where clinically appropriate, formal inter-reader reliability assessment, and pre-specified comparison with cross-sectional imaging, before CEUS perfusion features can be integrated into guideline-endorsed practice for selected pleura-based pulmonary lesions in STS [13,20].

## Figures and Tables

**Table 1:** CEUS perfusion patterns across pulmonary entities—comparison with published cohorts. Time to enhancement (PA vs. BA), extent of enhancement (marked vs. reduced), homogeneity of enhancement (homogeneous vs. inhomogeneous), and decrease in enhancement (rapid vs. late washout) are summarized for pleura-based soft-tissue sarcoma metastases (present study) and contrasted with pulmonary metastases and other lung pathologies from prior studies.

Underlying Disease	Soft Tissue Sarcoma	Pulmonary Metastases	Central Lung Cancer	Peripheral Lung Cancer	Lung Lymphoma	Obstructive Atelectasis	Acute Pneumonia	Granulomatous Disease	Organized Pneumonia
**Author**	Present study	Kroenig et al. [20]	Safai Zadeh et al. [26]	Findeisen et al. [27]	Trenker et al. [12]	Safai Zadeh et al. [26]	Linde et al. [28]	Safai Zadeh et al. [11]	Safai Zadeh et al. [10]
**No. of cases**	12	54	48	89	6	54	50	10	38
**Year**	2026	2024	2024	2019	2018	2024	2012	2021	2021
**Pattern of enhancement on CEUS**									
**TE: PA** **BA**	16.7% 83.3%	7.4% 92.6%	10.4% 89.6%	28.1% 71.9%	83.3% 16.7%	85.2% 14.8%	92.0% 8.0%	0% 100%	28.9% 71.1%
**EE: Marked** **Reduced**	75.0% 25.0%	68.5% 31.5%	8.3% 91.7%	59.5% 40.5%	100% 0.0%	n.a.	74.0% 26.0%	0% 100%	76.3% 23.7%
**HE: Hom** **Inhom.**	66.7% 33.3%	51.86% 48.14%	91.7% 8.3%	23.6% 76.4%	66.7% 33.3%	72.2% 27.8%	78.0% 22.0%	0% 100%	18.4% 81.6%
**DE: Rapid** **Late**	100% 0.0%	98.1% 1.9%	79.2% 20.8%	n.a.	50.0% 50.0%	27.8% 72.2%	n.a.	100% 0%	50.0% 50.0%

BA: bronchial arterial; CEUS: contrast-enhanced ultrasound; DE: decrease in enhancement; EE: extent of enhancement; HE: homogeneity of enhancement; Hom: homogeneous; Inhom: inhomogeneous; n.a.: not analyzed; PA: pulmonary arterial; TE: time to enhancement.

## Data Availability

The data presented in this study are available on request from the corresponding author due to privacy and ethical restrictions related to patient data.

## Abbreviations and Acronyms

B-LUS	B-mode lung ultrasound
BA	bronchial-arterial
CEUS	contrast-enhanced ultrasound
CI	confidence interval
CT	computed tomography
DE	decrease in enhancement
EE	extent of enhancement
EFSUMB	European Federation of Societies for Ultrasound in Medicine and Biology
HE	homogeneity of enhancement
ICU	intensive care unit
MRI	magnetic resonance imaging
NPAs	non-perfused areas
ÖGUM	Österreichische Gesellschaft für Ultraschall in der Medizin
PA	pulmonary-arterial
PET-CT	positron emission tomography–computed tomography
STS	soft tissue sarcoma
TE	time to enhancement
